# Soyabean Powder as a Novel Diluent in Tablet Formulation of Simvastatin

**DOI:** 10.4103/0250-474X.73909

**Published:** 2010

**Authors:** G. Swami, Khushboo Gupta, K. M. Kymonil, Shubhini Saraf

**Affiliations:** Department of Pharmaceutics, Faculty of Pharmacy, Babu Banarasi Das National Institute of Technology and Management, Lucknow - 227 105, India

**Keywords:** *In vitro* drug release, novel excipient, powder characteristics, simvastatin, soyabean nuggets

## Abstract

The present research paper introduces soyabean nuggets powder, as a novel excipient with nutraceutical value for tablets containing cholesterol lowering drug, simvastatin. Experiments were carried out to evaluate the suitability of soyabean nuggets powder as a diluent by incorporating in tablet formulation of simvastatin. The formulation was compared with the marketed product to determine its relative efficacy. Soyabean nuggets powder was found to be a promising diluent for tablets for both pharmaceutical and nutraceutical purposes. Simavastatin soya tablet showed acceptable pharmacotechnical properties and assay requirement.

Pharmaceutical excipients have a vital role in drug formulations[1]. Excipients are materials used in the formulation of pharmacologically active drugs; currently over a thousand such materials are used in marketed pharmaceuticals. They have a variety of roles including diluents, fillers, bulking agents, binders/adhesives, propellants, polymers and waxes; vaccine adjuvant also represents an excipient form. Excipients can be broadly divided into three categories; established, approved, new or novel, and essentially new excipients[2,3].

A novel excipient is a compound which has not been previously used or permitted for use in a pharmaceutical preparation. Essentially new excipients form an intermediate category and include substances resulting from a structural modification of an approved excipient, a recognized food additive (or cosmetic ingredient), a structurally modified food additive, or a constituent of an over the counter (OTC) medicine. Within industry there has been a recent surge of interest in the use and safety of established and new excipients in drug development[4,5].

Soyabean is high in protein and is considered equivalent to animal food in terms of the quality of the protein. It contains 43 g of protein per 100 g which is the highest among the pulses. It also contains 19.5 g of fat, 21 g of carbohydrate and provides 432 kcal per 100 g (Tables 1 and 2)[6]. Soyabean oil is one of the few common vegetable oils that contains a significant amount of alpha-linolenic acid, omega-3 fatty acids and omega-6 fatty acids[7]. Soy protein is associated with significant decrease in serum cholesterol, low density lipoprotein LDL (bad cholesterol) and triglyceride concentrations[8]. Soy phytoestrogens (isoflavones, genistein and daidzein) adsorbed on to soy protein are suggested as the agent reducing serum cholesterol levels. The benefits could potentially reduce the risk of atherosclerosis and other cardiovascular diseases[9–12]. Both the FDA and the AHA recommend 25 g of soy protein when incorporated into a diet that is low in cholesterol and saturated fat. FDA in 1999 permitted the health claim that soya intake can reduce cholesterol and thus help guard against diabetes and heart disease[13–15]. Clausen *et al*., reported that the treatment with Abacor^®^, a soy-based dietary supplement, reduces plasma concentrations of total and low-density lipoprotein cholesterol in statin-treated hypercholesterolaemic patients[16].

The aim of this study was to prepare tablets with soyabean nuggets powder as a novel nutraceutical excipient (diluent) containing simvastatin. The powder was evaluated for parameters like morphology, angle of repose, percentage compressibility, moisture capacity and ash value. The prepared tablets were evaluated for post compression parameters like hardness, friability, weight variation, uniformity of content, disintegration test, dissolution profile, stability studies and the formulations suitable to achieve our goal were determined.

**TABLE 1:** NUTRITIVE VALUE OF SOYABEAN AS COMPARED TO OTHER PER 100 G.

Pulses	Protein (g)	Fat (g)	Calcium (mg)	Carbohydrate (g)	Energy (kcal)
Bengal gram dhal	20.8	5.6	56	59.8	372
Green gram dhal	24.5	1.2	75	59.9	348
Lentil	25.1	0.7	69	59.0	343
Dry peas	19.7	1.1	75	56.5	315
Rajmah	22.9	1.3	260	60.6	346
Soya bean	43.2	19.5	240	20.9	432

**TABLE 2:** COMPOSITION OF SOYBEAN NUGGETS (NUTRILA)

Components	Quantity (%)
Protein	53.55
Fat	0.8
Moisture	8
Crude fibres	15
Carbohydrates	33.5
Ash	6
Calories (g/100 g)	290
Calcium content	0.20
Iron	0.008
Magnesium	0.27
Phosphorus	0.58
Potassium	2.80
Sodium	0.02

## MATERIALS AND METHODS

Simvastatin was a gift sample from Ranbaxy Laboratories limited (New Delhi, India). Soyabean nuggets (Nutrila) was procured from the local market, microcrystalline cellulose (avicel), polyvinylpyrollidone, magnesium stearate and talc used were of official quality. *In vitro* release chemicals (AR grade) were obtained from M/s S. D. Fine Chemicals, Mumbai, India).

### Soyabean powder and characterization of the powder:

The marketed soyabean nuggets were size reduced and sieved through a mesh (355 μm) and dried at 40° for 30 min. The powder was then used as the free flowing diluent for simvastatin tablet formulations (100 tablets). The powder parameters were evaluated and these were morphology, angle of repose, percentage compressibility, moisture capacity and ash value.

### Preparation of simvastatin soya tablets:

Tablets of soyabean powder and microcrystalline cellulose as diluents in different proportions containing simvastatin were prepared by wet granulation method using 4% polyvinyl pyrollidone in acetone as binder. The composition of different formulations used in the study is given in Table 3. In all the formulations soyabean powder was mixed with simvastatin and microcrystalline cellulose. The powders were blended and granulated with 4% PVP in acetone. The wet mass was passed though a mesh (aperture size 335 μm) and the granules were dried at 50° for 2 h. The dried granules were passed though a mesh (aperture size 180 μm), and these granules were lubricated with a mixture of talc and magnesium stearate (1:1.5). The lubricated granules were compressed at a compression of 1500 to 2500 kg using 3 mm round, flat and plain punches on a ten station tabletting machine (Mini Press-1, M/s Karnavati, Ahmedabad, India). Tablets of each composition were compressed (100 each) and their hardness, drug content and drug release characteristics were tested using required number of tablets for each test. The hardness of the tablets was determined using a Monsanto hardness tester.

**TABLE 3:** COMPOSITION OF SIMVASTATIN SOYA TABLETS

Ingredients* (mg)	Formulation code					
	F1	F2	F3	F4	F5	F6
Simvastatin	10	10	10	10	10	10
Soyabean	94	89	84	79	74	69
Microcrystalline cellulose	12	17	22	28	33	38
Polyvinyl pyrollidone	4	4	4	4	4	4
Magnesium striate	3	3	3	3	3	3
Talc	2	2	2	2	2	2

* All the quantities expressed are in mg/tablet. Formulation F4 was selected as the best and used for further studies.

### *In vitro* drug release studies[17]:

Drug release studies were performed using USP test apparatus (Apparatus I, 50 rpm, 37°; 900 ml) in pH 7.0 buffer solution containing 0.5% dodecyl sodium sulfate in 0.01 M sodium phosphate. Ten milliliters of the dissolution sample was taken after 30 min, suitably diluted with blank dissolution fluid and analyzed for simvastatin content by using Shimadzu 1400 UV spectrophotometer at 247 nm and compared with marketed formulation.

### Stability studies:

Stability studies were conducted on simvastatin soya tablets. One selected fabricated tablet batch was aluminum packaged and kept at 40° and 75% RH. Sample were withdrawn at 0, 15, 30 and 45 days to assess their stability with respect to their physical appearance, drug content and drug release characteristics.

## RESULTS AND DISCUSSION

**Fig. 1:**
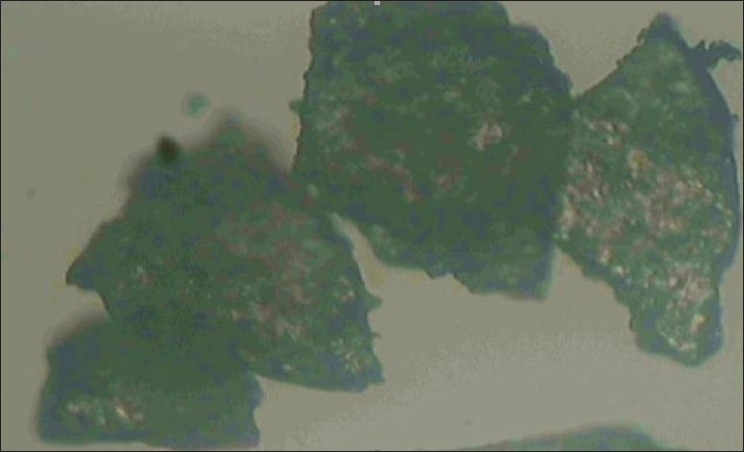
Photomicrograph of soyabean powder Photomicrograph showing rough flattened particles of soyabean nuggets powder, magnification 100×.

**Fig. 2:**
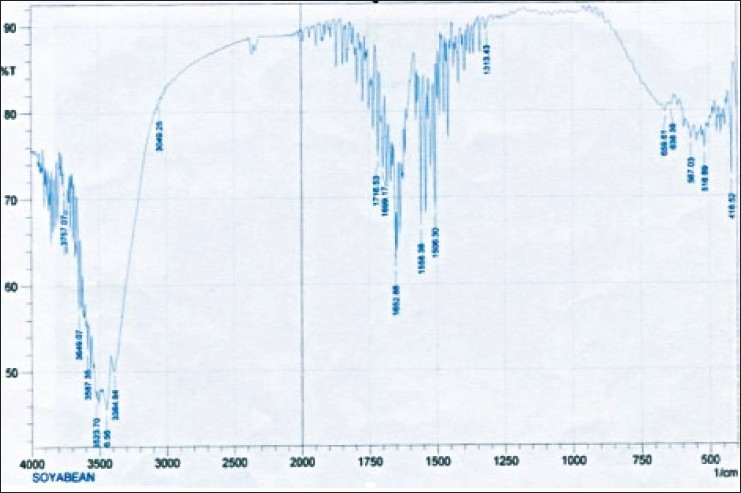
IR spectrum of soyabean powder IR Spectrogram of soyabean powder showing characteristic peaks

Microscopic examination of soyabean nuggets (powdered) showed rough and flattened particles (fig. 1). Angle of repose was determined by fixed-funnel method and was found to be 40.61±2.144°indicating poor flow properties. Other parameters such as bulk density (0.626±0.001 g/cm^3^), tapped density (0.667±0.014 g/cm^3^), hausner ratio (1.066), percentage compressibility index (6.259%), ash value (72.57%) and swelling capacity (24%) were also determined (Table 4). IR spectroscopic examination showed various characteristics peaks in the IR spectrum (Table 5, fig. 2). Since the powder was found to have poor flow properties, wet granulation method was used to improve the flow properties. The physical attributes of the tablets were found to be satisfactory. The tablets prepared were biconvex, pale yellow in color and had smooth texture without any cracks. Typical tablet defects, such as capping, chipping, and picking, were not observed. Results of other physical evaluations were found to be within an acceptable range. Weight variation was calculated as 4.87– 6.5% (range±7.5%), where average weight was 125.11 mg (n=20). Friability of the tablets was calculated in the range 0.2 to 0.9% (n=20), which was well within the acceptable range of 1% and indicates that tablet surfaces are strong enough to withstand mechanical shock or attrition during storage and transportation and until they are consumed (Table 6). It was found that with the increase in the quantity of soyabean nuggets powder in the tablet formulation friability increased which may be due to poor binding. But with the increase in the quantity of microcrystalline cellulose, tablets were less friable with higher drug release. Formulation (F-4) was found to shown highest percentage of drug release of 96.56% because of higher drug content. The assay results were in the range of 98.58±0.76% to 101.56±1.12%.

**TABLE 4:** CHARACTERSTICS OF SOYABEAN NUGGETS POWDER (NUTRILA)

Characterstics	Observed value
Morphology	Rough flattened
Angle of repose	40.61±2.144
Bulk density	0.63±0.001
Tapped density	0.68±0.014
Hausner ratio	1.066
Percentage compressibility index	6.26%
Ash value	72.57%
Swelling capacity	24%

**TABLE 5:** IR SPECTRUM OF SOYABEAN NUGGETS POWDER

Peaks (cm^−1^)	Groups	Remarks
3334.64	N-H	N-H Stretching
3049.25	CH	C-H Stretching
1716.33	CO	C-O Aliphatic acid
1556, 1558	NO	N-O Symmetric bending
1313.48	NO	NO_2_ (Nitro Compound)
638.11	CH	C-H Stretching

**TABLE 6:** EVALUATION OF SIMVASTATIN SOYA TABLETS

Parameter	F1	F2	F3	F4	F5	F6
Appearance	Bicovex tablets	Bicovex tablets	Bicovex tablets	Bicovex tablets	Bicovex tablets	Bicovex tablets
	yellowish colour	yellowish colour	yellowish colour	yellowish colour	yellowish colour	yellowish colour
Texture	Smooth	Smooth	Smooth	Smooth	Smooth	Smooth
Weight variation mg±SD	125 (8.18)	125 (7.153)	125 (7.26)	125 (6.09)	125 (7.38)	125 (6.27)
Hardness kg/cm^2^±SD	Less than 0.5	1.5–2 (0.25)	1.6–2 (0.70)	2–2.5 (0.25)	1–1.5 (0.25)	1.5–2 (0.40)
Drug content (%)±SD	99.75 (0.98)	99.85 (0.99)	99.91 (1.13)	101.56 (1.12)	98.58 (0.76)	99.63 (0.96)
Drug release % (after 30 min.)	92.23	89.92	91.33	96.56	95.66	96.01
Friability (%)	0.8	0.7	0.9	0.2	0.8	0.5

The values indicate mean ± SD. Standard deviation is between parentheses, n=3.

**Fig. 3:**
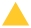
Comparative *in vitro* release profile of F4 and marketed formulation Pure drug (—
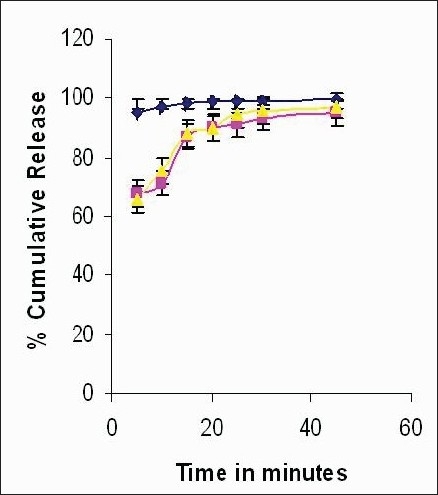
—), marketed formulation (—
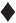
—) and simvastatin soya tablet (—
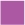
—)

The dissolution studies indicated that the formulations showed complete release of drug (89.33% to 96.01%) within 30 min compared to market sample which showed 90±3% drug release (fig. 3) which may be due to the uptake of water and swelling of soyabean powder leading to faster disintegration and release of drug. Thus use of superdisintegrants can also be avoided. A one way test of variance (ANOVA) was applied across all the formulations (F1 to F6). It was found that F2 and F4 were not significantly different on the basis of dissolution profile (Table 7). By comparing other parameters such as friability, hardness, and drug content it was found that F4 is the optimum formulation among all the formulations of soyabean- simavastatin tablet.

**TABLE 7:** DATA FOR ONE WAY TEST OF VARIANCE (ANOVA) ACROSS FORMULATIONS F1 TO F6

Comparison	p	q	*P*<0.05
F-4 vs F-2	6	4.867	Yes
F-4 vs F-3	5	4.648	Yes
F-4 vs F-1	4	4.323	Yes
F-4 vs F-5	3	3.795	Yes
F-4 vs F-6	2	2.777	Yes
F-6 vs F-2	5	4.648	Yes
F-6 vs F-3	4	4.323	Yes
F-6 vs F-1	3	3.795	Yes
F-6 vs F-5	2	2.777	Yes
F-5 vs F-2	4	4.323	Yes
F-5 vs F-3	3	3.795	Yes
F-5 vs F-1	2	2.777	Yes
F-1 vs F-2	3	3.795	Yes
F-1 vs F-3	2	2.777	Yes
F-3 vs F-2	2	2.777	Yes

**Fig. 4:**
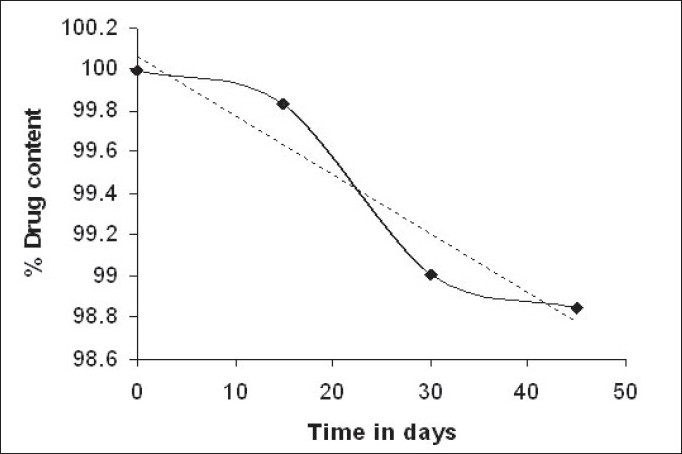
Stability profile for simvastatin soya tablets Remaining percentage of simvastatin content at 40°/75% RH after 15, 30 and 45 days

Stability study was carried out at 40°/75% RH for 45 days. At the end of the testing period, the simvastatin soya tablets were observed for change in physical appearance, analyzed for drug content and drug release characteristics. No visible changes in the appearance of simvastatin soya tablets were observed at the end of the storage period. The drug content was found to be 98.8 ± 0.67% (fig. 4).

The formulation of simvastatin soya tablet showed acceptable pharmacotechnical properties and assay requirements. It was concluded that crushed soya nuggets can act as a novel nutraceutical additive/excipient for simvastatin tablets providing bulk to the tablet as well synergistic effect for lowering serum cholesterol level.

## References

[1] Baldrik P (2000). Pharmaceutical Excipient development: The Need for Preclinical Guidance. Regul Toxicol Pharmacol.

[2] Shangraw R (1986). Developments in tablet excipients since 1960. Manuf Chem.

[3] Robertson MI (1999). Regulatry issues with excipients. Int J Pharmacol.

[4] Steinberg M, Borzelleza JF, Enters EK, Kinoshita FK, Loper A, Mitchell D (1996). A new approach to the safety assessment of pharmaceutical excipients. Regul Toxicol Pharm.

[5] Baldrik P, Swarbrick J, Boylan C (2000). Pharmaceutical Excipient testing: A regulatory and preclinical perspective. Encyclopedia of Pharmaceutical Technology.

[6] John M, Newton A, Karthikeyan M, Ramasamy C (2008). Soy-Protein: A treatment supportive Protein: An overview. Indian J Pharm Edu Res.

[7] Sacks FM, Lichtenstein A, Van Horn L, Harris W, Kris-Etherton P, Winston M (2006). Soy protein, isoflavones, and cardiovascular health: An American heart association science advisory for professionals from the nutrition committee. Circulation.

[8] Zhuo XG, Melby MK, Watanabe S (2004). Soy Isoflavone intake lowers serum LDL cholesterol: A meta-analysis of 8 randomized controlled trials in humans. J Nutr.

[9] Anderson JW, Cook-Newell ME, Johnston BM (1995). Meta-analysis of the effects of soy protein intake on serum lipids. N Engl J Med.

[10] Sirtori CR, Gianazza E, Manzoni C, Lovati MR, Murphy PA (1997). Role of isoflavones in the cholesterol reduction by soy proteins in the clinic. Am J Clin Nutr.

[11] Scheiber MD, Liu JH, Subbiah MT (2001). Dietary inclusion of whole soy foods results in significant reductions in clinical risk factors for osteoporosis and cardiovascular disease in normal postmenopausal women. Menopause.

[12] Anthony MS, Clarkson TB, Williams JK (1998). Effects of soy isoflavones on atherosclerosis: Potential mechanisms. Am J Clin Nutr.

[13] Sagara M, Kanda T, NJelekera M (2004). Effect of dietary intake of soy protein and isoflavones on cardiovascular disease risk factors in high risk middle: Aged men in Scotland. J Am Coll Nutr.

[14] Rivas M, Garay RP, Escanero JF, Cia P, Cia P, Alda JO (2002). Soy milk lowers blood pressure in men and women with mild to moderate essential hypertension. J Nutr.

[15] Lichtenstein AH (1998). Soy protein, isoflavones and cardiovascular disease risk. J Nutr.

[16] Clausan P, Lindhardsen J, Hoie L, Stender S (2004). Treatment with Abacor^®^, a soy-based dietary supplement, further reduces plasma concentrations of total and low-density lipoprotein cholesterol in statin-treated hypercholesterolaemic patients. Innov Food Sci Emerg Technol.

[17] Singla N, Gupta GD, Kohli K, Singla AK (2009). A discriminatory and biorelevant dissolution test method for simvastatin drug products. Dissolution Technologies November.

